# Micronutrient Differences in Conventionally and Organically Produced Foods

**DOI:** 10.3390/nu18010084

**Published:** 2025-12-26

**Authors:** Vaishnavi Balaji, Maja Chec, Raaga Brahmadevi, Steven Holladay, Krzysztof Czaja

**Affiliations:** 1 Department of Biomedical Sciences, College of Veterinary Medicine, University of Georgia, Athens, GA 30602, USA; vaishnavi.balaji@uga.edu (V.B.); maja.chec@uga.edu (M.C.); raaga.bramhadevi@uga.edu (R.B.); sdholl@uga.edu (S.H.); 2 Department of Biomedical Science, School of Veterinary Medicine, Ross University, Basseterre P.O. Box 334, Saint Kitts and Nevis

**Keywords:** micronutrients, organic farming, conventional farming, vitamins, minerals, polyphenols

## Abstract

Organic foods are often more expensive because the use of synthetic pesticides, herbicides, and fertilizers is prohibited, and production generally requires more labor. Consumers may feel that organically produced foods are healthier than conventionally produced; however, studies on nutritional value are mixed. This review examines existing reports of micronutrients, including vitamins, minerals, and other bioactive compounds, in plant- and animal-based foods from organic and conventional systems. A literature review was conducted using keywords related to micronutrients, organic farming, and conventional farming. Because the number of available studies was limited, no publication date restrictions were applied. After a preliminary screening of abstracts to determine their relevance to the study’s purpose, 120 articles were included. Reports are reviewed describing higher amounts of vitamin C, vitamin E, magnesium, iron, and polyphenols in organic produce and of certain animal products showing more antioxidants or fat-soluble vitamins. At the same time, many studies found no noteworthy differences, and some authors report higher nutrient levels in conventionally raised foods. Factors such as soil fertility, fertilizer use, and climate conditions may then influence micronutrient food content as much or more than farming methods. While organic foods may sometimes provide enhanced micronutrient levels, the overall evidence from existing literature does not support a consistent nutritional advantage associated with organic production.

## 1. Introduction

Organic food products have been more prominent in the interest of consumers due to the farming and raising practices of organic produce and livestock. These practices include the forbidding of chemically synthesized fertilizers, genetically modified organisms, and antibiotics, with a greater focus on ecological principles and natural principles [1]. Many people are willing to pay premium prices for products with organic labels, assuming they are healthier and better to consume. However, whether these methods consistently improve the micronutrient content of foods remains uncertain. Unlike macronutrients, which supply energy in large amounts, micronutrients are needed only in small quantities yet are critical for biological processes. Vitamins, minerals, polyphenols, and trace elements support enzyme activity, regulate immune defenses, and maintain tissue integrity [2,3]. Because of their essential role in human health, even small changes in micronutrient levels can have meaningful effects. This review examines differences in the micronutrient composition of plant- and animal-based foods produced under organic and conventional systems, with a focus on their potential impact on human health.

## 2. Methods

This review was conducted with the aim of providing a non-exhaustive but thorough report on micronutrient parameters in organically and conventionally produced food products followed by an extensive literature search.

The literature search was based on researching the given micronutrient, finding relevant studies demonstrating parameters in organic and conventional food products, and researching potential explanations for the outcome of the found results. Studies and prior reviews were found by the use of the PubMed and Google Scholar databases with search key terms relating to food product studies corresponding to high levels of the micronutrient of interest or synthesis processes of the micronutrient in the food product (i.e., Smirnoff–Wheeler pathway). Over 120 articles were initially screened, but only those directly relevant to the micronutrient outcomes of interest were included in the final review. Despite the search including majority of articles from the years 2000–2025, due to the demand for specification of the data and limited studies conducted, overall, there was no restriction regarding the timeline of the data or exclusion criteria as the finding of a rational explanation of the results was prioritized. This review focused on quantitative studies as well as review papers previously published on the topic. All included publications were written in English.

## 3. Micronutrients

### 3.1. Vitamins

Vitamins are classified as organic molecules and are subdivided into two groups: water-soluble and lipid-soluble vitamins. Water-soluble vitamins consist of vitamin C and all forms of vitamin B. The lipid-soluble vitamins consist of vitamins A, D, E, and K. There are differential roles of vitamins; however, all involve the support of human metabolism and function [4].

#### 3.1.1. Vitamin A

Vitamin A is a liposoluble vitamin and antioxidant agent that exists in two categories of compounds: retinoids and provitamin A carotenoids [5]. Proper functioning of the immune system, tumor-suppressing mechanisms, cell differentiation, organogenesis, vision, male and female reproductive systems, and embryonic development require vitamin A intake [6,7,8,9]. Additionally, carotenoids, especially β-carotene, act as antioxidants, reducing oxidative damage to essential macromolecules and development of chronic diseases [10,11,12]. β-carotene is the primary dietary provitamin A for plant-based diets. There is income-related skewing toward increased dietary plant components for many low- and middle-income populations, making considerations of β-carotene content in organic and conventional produce important for health considerations [13]. Presently available data depicting levels of vitamin A in conventional and organic produce cultivars presents ambiguous conclusions [1]. While some studies report higher β-carotene in organic red and pickled bell peppers [14,15] and in organic Chinese mustard and swamp cabbage [16], others find higher levels in conventionally grown carrots [17] or total carotenoid values in paprika peppers [18], and several report no significant differences [19,20,21,22]. The variation in results of the studies and the parameters recorded can be potentially associated with the influence of environmental stresses and fertilization of the plants in food production systems. Fertilizer amount and form, particularly total nitrogen and the NO_3_: NH_4_ ratio, can either boost or inhibit β-carotene. Higher N is linked to greater carotenoids in some studies [17,23], yet increased total N with lower NO_3_: NH_4_ inhibited carotenoid accumulation in carrot callus [19]. Excess ammonium may also raise reactive oxygen species (ROS) and degrade carotenoids [24]. Moreover, stresses like herbivory, which can suppress the methylerythritol phosphate (MEP) carotenoid biosynthesis pathway and lower β-carotene by as much as 40% [25,26], along with drought or irrigation differences [27], alter carotenoid synthesis and may help explain inconsistent results across studies. Table 1a summarizes the quantitative values of β-carotene in plant-based foods from the analyzed literature. Studies suggest the animal’s diet largely drives differences in vitamin A between organic and conventional products. In Egypt, organic eggs contained significantly higher vitamin A than conventional eggs [28]. Organic beef showed 53% more β-carotene overall, with longissimus thoracis (LT) muscle levels 351% higher, likely due to grass-rich diets [29]. While beef is not a major vitamin A source, β-carotene can extend shelf life by protecting against oxidation [9]. In salmon, carotenoid levels did not differ significantly, although organic fish had a more diverse carotenoid profile, reflecting the potential effect of a carotenoid-rich feed [30]. Organically reared Krškopoliće pigs fed alfalfa hay had higher vitamin A in backfat, linked to greater unsaturated fatty acids and lipid oxidation [31]. Overall, feed composition and bioaccumulation from organic plants may explain these differences, but more research is needed to clarify the role of diet. Moreover, carotenoid bioavailability, including that of β-carotene, is influenced by the surrounding food matrix and dietary fat, which together enhance micelle formation and intestinal uptake [32]. Free-form carotenoids are more accessible than those bound within plant tissues, but food processing, such as cooking and boiling, can increase bioavailability by disrupting cell walls, whereas fiber and other components can hinder carotenoid release [32]. Hence, it is important to understand that total carotenoid levels in a crop may not reflect the amount actually absorbed, which affects whether differences between these farming systems have a meaningful health impact. Quantitative differences in Vitamin A in these animal-based products are demonstrated in Table 1b.

#### 3.1.2. Vitamin B

There have been few studies comparing vitamin B levels in organic versus conventional foods, and those that exist span long periods during which the definition of “organic” has changed, potentially leading to discrepancies in the results. For instance, one study comparing reviews from 1924 to 1994 showed no meaningful differences in Vitamin B content between organic and conventional potatoes, vegetables, and cereals. However, this 70-year time span includes considerable conventional production changes and variation in sources of samples [33]. The differences between organic and conventional foods also become more complicated when considering the various types of vitamin B. A separate comparison of the eight different types of vitamin B is provided in Table 2.

#### 3.1.3. Vitamin C

Vitamin C (ascorbic acid) is a water-soluble antioxidant essential for humans who cannot synthesize this vitamin due to lack of the L-gluconolactone oxidase gene [51,52,53,54]. Ascorbic acid must therefore be obtained from dietary sources such as citrus fruits, kiwifruit, berries, leafy greens, tomatoes, and potatoes [54]. In the human body, vitamin C supports innate and adaptive immunity (enhancing B- and T-cell activity, neutrophil protection, and epithelial barrier function), protects DNA and proteins from oxidative stress, and is believed to reduce risk of chronic diseases such as atherosclerosis and cancer [51,52,55,56]. Research comparing vitamin C in organic and conventional produce shows mixed results but suggests a tendency for higher concentrations in organic fruits and vegetables. This is most consistent in leafy greens, where organic spinach, lettuce, and swamp cabbage had higher ascorbic acid [16,33,57,58]. Citrus fruits such as mandarins and grapefruit, as well as organic tomatoes and potatoes, also showed higher vitamin C in several studies [59,60,61,62,63]. In contrast, studies on Barbados cherry, persimmon, strawberries, broccoli, and some blueberries showed equal or in some cases higher vitamin C levels [64,65,66]. Antioxidant activity was lower in organic blueberries due to their larger fruit size, which correlates with reduced ascorbic acid concentration, and to reduced environmental stress conditions since these organic blueberries were grown under conditions viewed as lower stress [66]. Antioxidant activity often paralleled ascorbic acid levels, with higher activity observed in organic tomatoes, mandarins, and grapefruit [59,61,62]. Quantitative values of ascorbic acid content recorded in the reviewed literature are summarized in Table 3.

One explanation for higher vitamin C in organic foods is the carbon–nitrogen balance theory, which suggests that excess nitrogen fertilizer in conventional systems correlates with reduced non-structural carbohydrates (e.g., glucose), slowing the Smirnoff–Wheeler pathway that synthesizes ascorbic acid [52,67,68]. In addition, organic crops face greater biotic and abiotic stresses, which activate defense pathways such as ascorbate peroxidases that use vitamin C to neutralize ROS [62,69,70]. These stresses often include limited nitrogen availability and poor weed suppression, which restrict plant growth [71]. Moreover, organic crops usually utilize fewer pesticides, if any, leading to problems with pest and disease control, which can also yield losses in organic crop production [71].

Environmental factors, including sunlight, further affect synthesis: summer harvests yield higher ascorbic acid due to light-driven upregulation of Smirnoff–Wheeler pathway genes [52,72]. Together, these mechanisms may help explain instances where organic foods show higher vitamin C, with variability reflecting differences in cultivation practices and growing conditions.

#### 3.1.4. Vitamin D

Vitamin D is a lipid-soluble vitamin that functions to maintain and regulate calcium levels in the body [73]. It is synthesized in the skin via UV-B exposure or obtained from foods such as salmon, dairy, and fortified products [74]. Vitamin D circulates as 25(OH)D3, which is converted in the kidneys to calcitriol, the active hormone regulating calcium and phosphorus metabolism [75]. The biosynthesis of vitamin D in plants is less known, which has raised concerns about different populations that depend on a plant-based diet having a high prevalence of vitamin D deficiency. Moreover, there is no clear data comparing Vitamin D content across production systems, making it presently uncertain whether differences exist. In terms of animal-based products, Vitamin D concentration is high in the flesh of oily fish [76]. For organic and nonorganic animal and dairy content, there is a mix of published results. Wild salmon contain up to 4 times more vitamin D3 than farmed salmon, although levels again vary by region (e.g., Baltic vs. Irish waters) and aquaculture diet supplementation often fails to replicate wild concentrations [77]. 25-hydroxyvitamin D3, the circulating form, has been reported as higher in farmed fish, although these results are again inconsistent [74]. Among terrestrial farm animals, feed and UVB exposure strongly influence vitamin D content. Organic and free-range eggs show significantly higher vitamin D3 and 25(OH)D3 compared to caged hens [74], while milk shows little difference, with some studies finding higher vitamin D3 in conventional milk [76]. Overall, fatty fish remains the most reliable dietary source of vitamin D [75]. Table 4 demonstrates the quantitative differences in vitamin D in organic and conventional animal-based foods analyzed.

#### 3.1.5. Vitamin E

Vitamin E (tocopherols and tocotrienols) is a fat-soluble vitamin and a key antioxidant, protecting membrane lipids and lipoproteins from oxidative damage while also supporting immune function [78]. No difference was found in cabbage over three years [79], while some reviews reported lower vitamin E in conventional crops using synthetic nitrogen fertilizers, which possibly reduced vitamin production [80], and higher levels in 62% of organic–conventional crop pairs [81]. Conversely, conventionally produced sunflower seed oil showed higher α-tocopherol [82]. In terms of animal-based products, a study in Krškopoliće pigs, Slovenia’s native breed, found that organic pigs had lower vitamin E but higher vitamin A in fat, likely because vitamin E was depleted protecting polyunsaturated fatty acids (PUFAs) [31]. In contrast, organic beef contained 24% more tocopherol than conventional beef, which was attributed to grass-rich diets higher in vitamin E [29]. These mixed findings suggest that fertilizer use, diet, and farming practices may influence vitamin E content in meat, but more research on confounding factors needs to be conducted. Table 5 provides a summary of the quantitative differences in vitamin E levels in the food products analyzed.

#### 3.1.6. Vitamin K

Vitamin K is a fat-soluble vitamin essential for blood clotting, bone health, and cardiovascular protection through its role as a cofactor for γ-glutamyl carboxylase [83,84,85]. The two main forms are K1 (phylloquinone), found in plants, and K2 (menaquinones), found in animal products and synthesized by gut bacteria, although poorly absorbed [83,86,87,88]. Research directly comparing organic and conventional crops for this vitamin is quite limited, but cultivation practices have been suggested as potentially influencing phylloquinone levels. Fertilization methods and stress exposure are known to affect vitamin synthesis in plants, as shown in micronutrients other than vitamin K [89,90,91]. Vitamin K1 bioavailability depends on dietary fat, bile salts, digestive efficiency, and the food matrix, as plant cell walls can limit absorption [32,90,92,93]. Cooking and mechanical processing can improve the release of vitamin K1 from leafy greens, while the presence of other nutrients or anti-nutrients may further affect vitamin uptake, similar to what has been seen for vitamin A [32]. Consequently, measured differences in total K1 between organic and conventional produce may not directly translate to differences in physiological availability. Overall, while vitamin K1 is critical for individuals on plant-based diets, especially vegans who lack K2 sources, more studies are needed to establish whether organic produce provides different K1 levels.

#### 3.1.7. Vitamin Summary

Vitamin parameters in both plant-based foods and animal-based foods are dependent on various factors, resulting in different trends observed in conventional and organic production systems. Although some vitamins—such as vitamin C- often show consistent patterns, additional studies are needed to determine whether one production system offers a definitive advantage. Studies reporting no statistically significant differences between organic and conventional production were included in the literature review and were referenced when creating the overall discussion. However, to maintain clarity, Table 6 was limited to studies showing a clear organic or conventional advantage for specific crop–nutrient comparisons. As a result, the findings presented in the table reflect individual study outcomes rather than consistent or generalizable trends and should be interpreted as context-dependent rather than evidence of nutritional superiority.

### 3.2. Minerals

Minerals are essential for bone strength, muscle health, water balance, and immune function. Macrominerals (calcium, phosphorus, magnesium, sodium, potassium, chloride, sulfur) are needed in larger amounts, while trace minerals (iron, zinc, copper, manganese, selenium, iodine) are required in smaller amounts [94,95]. Research comparing organic and conventional crops has to date shown mixed results but suggests a general trend of higher mineral content in organic produce, particularly iron, magnesium, phosphorus, and zinc, although manganese is often lower. However, some studies have suggested that conventional crops may have higher nutrient levels due to greater nitrogen availability, resulting from increased chemical fertilizer use [96]. Conversely, organic wheat grains have shown higher nitrogen uptake due to the active soil biology supported by organic soils [97]. While organic crops may have slightly higher zinc concentrations, the amount that the body can absorb depends on inhibitors such as phytates and fiber, which can bind zinc and limit uptake, and this is similar for other minerals such as iron and calcium. In other words, bioavailability depends on food matrix characteristics and processing methods, such as cooking, fermentation, or germination, that decrease antinutrient levels and can help enhance intestinal absorption [98,99]. Organic farming practices such as soil enrichment, crop rotation, and use of biofertilizers are likely to contribute to these differences, while environmental and seasonal factors may play a more major role [100,101,102]. Table 7 provides an overview of mineral content similarities and differences in selected crops grown under organic and conventional systems.

#### Heavy Metals

Heavy metals (such as Cd, Pb, As, and Cr) are environmental pollutants or natural constituents that can be toxic to the human body when consumed, causing gene mutations, DNA damage, and inducing apoptosis. There are various factors that can increase the presence of metals, but here we will specifically focus on agricultural activities, particularly on how differences in farming practices can lead to higher levels of these metals being consumed. For conventional farming, additional elements and nutrients may be added through fertilizers, leading to the accumulation of both pesticides and heavy metals in crops [80]. Moreover, meta-analyses have shown that organic crops contain, on average, about 48% less cadmium (Cd) than conventional ones, suggesting a potential dietary advantage, though the health impacts need to be examined in the long term [109]. Moreover, an assessment of toxic metal concentrations in the five most-consumed vegetables (tomato, potato, onion, lettuce, carrot) in the U.S. showed that the mean metal levels were higher for four of the five toxic metals in conventional foods, with particularly high amounts of cadmium and lead, as shown in Table 8 [110]. The study also revealed that the mean-to-MAC ratio was highest for cadmium in potatoes at 34%. Although cadmium and lead levels were lower in organic vegetables than conventional ones, all mean concentrations reported in Table 8 remain well below established safety thresholds (e.g., maximum allowable concentrations (MAC): Cd = 50 µg/kg). These findings indicate that, while organic production may modestly reduce exposure to these metals, the absolute dietary risk from the vegetables examined is low. Nevertheless, chronic dietary intake and cumulative exposure across multiple food sources should be considered when assessing overall risk. Moreover, a 2012 literature review by Smith-Spangler et al. [111] similarly reported that a majority of studies found lower Cd concentrations in organic foods compared with conventional ones. These differences are thought to arise mainly from fertilizer composition, particularly the heavy metal content in mineral phosphate fertilizers used in conventional systems, as well as soil and agronomic conditions such as pH, liming, and crop rotation [109].

### 3.3. Polyphenols

Polyphenols are micronutrients and the largest group of phytochemicals naturally occurring in plants, which are the major source of human consumption. Their high antioxidant and anti-inflammatory activities help prevent the development of cancers, cardiovascular diseases, and neurodegenerative diseases [1,112]. Despite consistent lower use of synthetic pesticides in organic agriculture, which can increase phenolic synthesis, multiple studies provide inconclusive data on the polyphenol content of raw conventional and organic vegetables and fruits [1]. Polyphenols contribute to plant defense under unfavorable growing conditions, potentially explaining the higher polyphenol levels that have been reported in some organic cultivars [113]. However, Ponder (conventional honeysuckle blueberries) contained higher levels of total polyphenols due to differences in annual climatic conditions, such as humidity, sunlight, and temperature, which influenced flowering time [114]. This demonstrates a study of ambiguity that may result from climatic conditions, on which these bioactive ingredients depend. Despite variances in antioxidant capacity based on the type of vegetable sampled (e.g., potato, broccoli), polyphenols in conventionally grown vegetables appear to be more stable compared with the sometimes-greater amounts found in organically produced crops [115]. Furthermore, regardless of these varying stress conditions, other factors such as genetic makeup, climate, and heat treatment methods influence polyphenol levels, resulting in what presently remains an indefinite claim that organic vegetables have been reported to contain greater nutritional value to consumers. For example, the phenolic profile in sorghum grains was significantly affected by genotype and growth temperature as the study examined 6 different genotypes of sorghum and the impact of changing growth temperatures. In tomatoes, the genotype ‘Kalvert’ stood out for accumulating higher amounts of chlorogenic acid, caffeic acid, rutin, myricetin, quercetin, and naringenin compared to other genotypes [116]. In addition, when examining different regions, they found that the polyphenol content differed between the cities in Extremadura and Navarra. The environment influenced polyphenol composition as well, with higher chlorogenic and ferulic acids but lower rutin levels found in Extremadura than in Navarra in 2012 [116]. Yearly variation can also play a role, as seen in broccoli where phenolic levels differed between the first and second year, leading these authors to hypothesize that in year one, there could have been increased stress which caused a general rise in total phenolic content through the metabolic pathway. Heat treatments can have different effects depending on the crop as strawberries showed an increase in total phenolic compounds under high temperature, while sweet potato leaves significantly decreased in total phenolic contents [116]. Additionally, heating and salt stress reduced polyphenol levels in vegetables including kale, green beans, and tomatoes, since osmotic imbalance occurs when water is lost, leading to higher polyphenol loss [117]. Therefore, given the wide variation caused by environmental, genetic, and processing factors, the polyphenol content and antioxidant capacity of organic produce alone do not appear to substantiate its higher market price.

## 4. Discussion

Overall, the findings of this review indicate that differences in micronutrient concentrations between organic and conventional foods are inconsistent and context-dependent. Rather than reflecting a uniform advantage of one production system, observed patterns appear to arise from a combination of biological, environmental, and management-related factors that cause differences in concentration of nutrients among different or even the same crops. This complexity helps explain the vast heterogeneity reported across studies and warns against interpreting isolated differences as evidence of nutritional superiority.

For antioxidant-related micronutrients, including vitamin C, carotenoids, and polyphenols, concentrations are strongly influenced by plant stress physiology rather than production system alone. Factors such as crop genotype, light exposure, and biotic or abiotic stress regulate key biosynthetic pathways, meaning that higher levels reported in some organic crops may reflect stress-induced metabolic responses. For example, organic spinach and tomatoes contained approximately 1.5 times higher ascorbic acid concentrations than their conventional counterparts (405 vs. 258 mg kg^−1^ and 400 vs. 279 mg kg^−1^, respectively), consistent with increased Vitamin C production through the Smirnoff–Wheeler pathway under conditions of limited nitrogen availability and elevated oxidative stress. In contrast, carotenoid accumulation exhibited crop-specific and sometimes opposing trends. Organic red bell peppers showed substantially higher β-carotene levels (295 vs. 85 mg kg^−1^), whereas conventionally grown carrots contained higher concentrations (45.0 vs. 33.1 mg kg^−1^). These varied quantitative patterns suggest that carotenoid content is affected by factors other than a consistent effect of organic cultivation.

In contrast, B vitamins and vitamin E appear to be more closely linked to soil nutrient availability and fertilizer composition, which vary widely both within and between organic and conventional systems. Organic fertilizers were associated with higher concentrations of certain B vitamins in leafy greens, while conventionally produced sunflower seed oil exhibited higher α-tocopherol levels, highlighting how nutrient form and availability may influence synthesis differently across crops. Mineral micronutrients, including iron, zinc, magnesium, and manganese, further illustrate the influence of confounding environmental factors. Their uptake is strongly regulated by soil chemistry, pH, organic matter content, and microbial activity, all of which differ across growing regions and management practices. As a result, studies conducted in different soils or climates frequently report diverse mineral profiles for the same crop, even when comparing similar production systems.

Animal-based foods demonstrated additional layers of complexity, particularly for fat-soluble vitamins such as vitamins A, D, and E. For these examples, nutrient concentrations were strongly influenced by feed composition, pasture access, sunlight exposure, and bioaccumulation. For example, organically reared beef contained 53% more β-carotene, likely due to pasture-based diets, whereas eggs showed slightly higher vitamin A levels under conventional production. Differences in vitamin D were largely species-specific rather than dependent on farming type. Organic salmon showed much higher vitamin D concentrations than farmed fish, likely due to greater exposure to natural diets and sunlight, whereas cow’s milk showed little variation between organic and conventional systems. This highlights that, for animal-based foods, nutrient content often reflects feeding and environmental conditions alongside the type of farming.

While we were able to target some of the confounding factors that these studies have, there are some limitations to the present review, which need to be considered when interpreting the results. First, the number of studies directly comparing micronutrient levels between organic and conventional foods is limited, and much of the available data focus on specific crops rather than a wide range of food types. Moreover, there is considerable variation in terms of study design and environmental conditions which all reduce cross-study reliability. In addition, definitions of “organic” have evolved over time, meaning that older studies may not align with current certification standards. Furthermore, differences in how nutrient concentrations are reported, such as on a fresh or dry weight basis, may further complicate comparisons and meta-analyses. Finally, by focusing primarily on micronutrient content, this review does not fully address other outcomes such as pesticide residues, heavy metal accumulation, and environmental sustainability, which are also important components of food quality and safety.

In terms of future directions, this area of food knowledge would greatly benefit from studies designed to control for external confounding factors and with aims focused on clarifying how environmental and biological variables influence nutrient concentration and overall food quality. For instance, examining factors such as climate and genotype variety would help identify optimal conditions for maximizing nutrient levels in both plant- and animal-based products [118]. It will also be important to analyze environmental factors such as soil health and microbiome dynamics to better visualize the mechanisms driving micronutrient variation across the two farming systems [80]. In addition, more large-scale studies should optimally be conducted to evaluate the long-term health impacts of organic foods, including nutrient bioavailability, to determine whether differences in micronutrient content are truly significant from a health perspective [119]. Finally, future research should also include macronutrients, as these are required in large amounts to sustain energy production and maintain physiological processes.

In conclusion, the collective body of existing literature suggests that while organic production may confer certain advantages, particularly in antioxidant content and lower contaminant levels, the overall nutritional superiority of organic foods cannot be generalized and must be interpreted within the context of crop type, environment, and specific management practices.

## Figures and Tables

**Table 1:** (a) Comparison of quantitative parameters of β-carotene levels in plant-based foods from organic and conventional production systems. (b) Comparison of quantitative parameters of various Vitamin A derivatives in animal-based foods from organic and conventional production systems.

Food Product	Organic	Conventional	Source
(a)			
Red Bell pepper	295 ± 8 mg·kg^−1^ (fresh weight)	85 ± 5 mg·kg^−1^ (fresh weight)	[14]
Pickled Bell pepper	8.7 ± 2.3 mg·kg^−1^ (fresh weight)	7.0 ± 2.1 mg·kg^−1^ (fresh weight)	[15]
Carrot	33.1 ± 6.3 mg·kg^−1^ (fresh weight)	45.0 ± 7.6 mg·kg^−1^ (fresh weight)	[17]
Paprika peppers	58.5 ± 2.5 mg·kg^−1^ (dry weight)	53.2 ± 3.0 mg·kg^−1^ (dry weight)	[18]
(b)			
Egg	1.54 ± 0.0672 mg·kg^−1^ (vitamin A)	1.35 ± 0.0483 mg·kg^−1^ (vitamin A)	[28]
Beef	0.60 ± 0.06 mg·kg^−1^ (β-carotene)	0.39 ± 0.07 mg·kg^−1^ (β-carotene)	[29]
Salmon	8.8 ± 1.24 mg·kg^−1^ (total carotenoids)	7.8 ± 1.37 mg·kg^−1^ (total carotenoids)	[30]
Pork (backfat)	0.675 mg·kg^−1^ (vitamin A)	0.600 mg·kg^−1^ (vitamin A)	[31]

**Table 2:** B vitamin reports in organic and conventional foods, including their functions, observed differences, explanations, and sources.

Vitamin	Function	Differences	Explanation	Sources
B1	Cofactor for cerebral metabolism	Crops grown with organic fertilizers, such as spinach, green gram, and wheat, contain higher thiamine levels.	While organic fertilizers generally enhance thiamine levels through nutrient-rich soil and microbial activity, nitrogen-based fertilizers can also increase B1 concentrations.	[33,34,35]
B2	Antioxidant properties	Organic Chinese mustard and swamp cabbage demonstrated higher levels of riboflavin.	Soil quality and nutrient-rich organic fertilizers may be potential reasons, but further research needs to be conducted.	[16,35,36]
B3	Cell respiration and energy metabolism	Data limited; unclear differences in plants.		[37]
B5	Energy generation, fatty acid synthesis	The limited number of published studies makes it difficult to analyze plant-based foods’ concentrations of vitamin B5.		[38]
B6	Coenzyme in 150+ reactions, antioxidant	Data limited	Absorption higher when natural B6 is consumed; effect of organic farming is not well quantified.	[39]
B7	Coenzyme in Krebs cycle	Natural soil biotin, a biofertilizer with organic matter-degrading enzymes, improved the resilience of Chinese cabbage grown in high-stress conditions.	Organic fertilizers increase microbial activity, enzyme content, and enhance plant vitamin content.	[35,40,41,42]
B9	DNA synthesis, methylation	Organic eggs show higher total folate.	Organic practices, such as pasture access and naturally fertilized fodder, help improve soil health and support folate retention. Humic/folic acids also mitigate plant stress and preserve nutrients.	[43,44,45,46]
B12	Fatty acid metabolism, methylation	Organic plants may absorb more B12 via organic fertilizers, limited data on meat	Organic fertilizers, such as manure, provide microbial vitamin B12 that enhances plant uptake. Since B12 is largely excreted in feces, it can re-enter the food chain through bioaccumulation, leading to higher B12 levels in organically raised meat.	[38,47,48,49,50]

**Table 3:** Comparison of quantitative parameters of vitamin C + ascorbic acid levels in animal-based foods from organic and conventional production systems.

Food Product	Organic	Conventional	Source
Spinach	404.8 ± 6.16 mg kg^−1^ (fresh weight)	257.5 ± 6.12 mg kg^−1^ (fresh weight)	[57]
Lettuce (*Lactuca sativa*)	0.239 mg kg^−1^	0.200 mg kg^−1^	[58]
Mandarin Oranges	419 ± 25 mg L^−1^	366 ± 18 mg L^−1^	[59]
Grapefruit	41.8 mg 100 g^−1^	39.2 mg kg^−1^	[61]
Tomato	400 ± 0.8 mg kg^−1^	279 ± 3.5 mg kg^−1^	[62]
Potato	105.4 ± 39.1 mg kg^−1^	80.9 ± 20.8 mg kg^−1^	[63]
Persimmon	11.85 ± 0.48 mg 100 g^−1^	11.99 ± 0.33 mg 100 g^−1^	[64]
Strawberry	30.74 ± 3.68 mg kg^−1^	42.45 ± 4.78 mg kg^−1^	[64]

**Table 4:** Comparison of quantitative parameters of vitamin D3 levels in animal-based foods from organic and conventional production systems.

Food Product	Organic	Conventional	Source
Salmon	24.9 µg/100 g	6.05 µg/100 g	[76]
Eggs	1.45 ± 0.45 µg/100 g	1.31 ± 0.45 µg/100 g	[74]
Milk	0.008 µg/100 g	0.010 µg/100 g	[74]

**Table 5:** Comparison of quantitative parameters of vitamin E in plant-based and animal-based foods from organic and conventional production systems.

Food Product	Organic	Conventional	Source
Sunflower Seed Oils	30 ± 0.01 mg kg ^−1^	90 ± 0.02 mg kg ^−1^	[82]
Pork (backfat)	11.9 mg kg ^−1^ (vitamin E)	14.3 mg kg ^−1^ (vitamin E)	[31]
Beef	2730 ± 0.14 mg kg ^−1^	2190 ± 0.08 mg kg ^−1^	[29]

**Table 6:** Summary of food products that contain higher levels of the given vitamin in discussion based on literature reviewed. Statistical significance (*p*) was based on the study conducted in the given source. Non-significant differences are demonstrated with “n.s.” Statistical significance is reported as defined by the original studies and reflects the analytical methods used by each set of authors. Because study designs differ across studies, these significance values should not be interpreted as directly comparable across the food items or production systems.

Food Item	Micronutrient	Results	Number of Studies	Statistical Significance
**Plant Based Foods**				
Red Pepper	**Vitamin A**	Organic	[14,15]	*p* < 0.001
Pickled Bell Pepper		Organic	[15]	*p* < 0.05
Chinese Mustard		Organic	[16]	*p* < 0.05
Swamp Cabbage		Organic	[16]	*p* < 0.05
Carrot		Conventional	[17]	*p* < 0.05
Paprika Peppers		Organic	[18]	*p* < 0.05
Carrot	**Vitamin B1**	Organic	[35]	n.s.
Spinach		Organic	[35]	
Green Gram		Organic	[35]	
Wheat		Organic	[35]	
Swamp Cabbage	**Vitamin B2**	Organic	[16]	*p* < 0.01
Chinese Mustard		Organic	[16,57]	*p* < 0.05
Carrot	**Vitamin B1**	Organic	[14]	n.s.
Spinach		Organic	[14]	
Green Gram		Organic	[14]	
Wheat		Organic	[14]	
Swamp Cabbage	**Vitamin B2**	Organic	[58]	*p* <0.01
Chinese Mustard	**Vitamin B2**	Organic	[58]	*p* <0.05
Spinach	**Vitamin C**	Organic	[57]	*p* < 0.001
Lettuce		Organic	[58]	n.s.
Swamp Cabbage		Organic	[16]	*p* < 0.05
Mandarin		Organic	[59]	*p* < 0.005
Grapefruit		Organic	[61]	*p* ≤ 0.05
Tomato		Organic	[62]	*p* < 0.05
Potato		Organic	[63]	n.s.
Persimmon		Conventional	[64]	n.s.
Strawberry		Conventional	[64]	*p* < 0.05
Broccoli		Organic	[65]	n.s.
Blueberry		Conventional	[66]	*p* < 0.05
Sunflower Seed Oils	**Vitamin E**	Conventional	[82]	*p* < 0.05
**Animal Based Foods**				
Egg	**Vitamin A**	Conventional	[28]	*p* < 0.01
Beef		Organic	[29]	*p* < 0.05
Salmon		Organic	[30]	n.s.
Pork		Organic	[32]	*p* < 0.05
Salmon	**Vitamin D**	Organic	[76]	
Eggs		Organic	[74]	n.s.
Milk		Conventional	[74]	
Pork	**Vitamin E**	Conventional	[32]	*p* < 0.05
Beef		Organic	[29]	*p* < 0.001

**Table 7:** Qualitative comparison of mineral content in selected crops grown under organic and conventional systems.

Crop	Higher in Organic	Higher in Conventional	Sources
Beetroot and Potatoes	Iron and magnesium		[103]
Sweet Potatoes	Calcium, Potassium, Phosphorus, Copper, Iron, Zinc, Manganese	Magnesium, Sodium	[104]
Corn grain	Phosphorus, magnesium, potassium	Sulfur, manganese, calcium, zinc	[100,105]
Tomatoes	Potassium, calcium, zinc, copper, manganese, sodium	Manganese and chromium	[9,102,106]
Lettuce	Chromium, copper, iron, potassium, magnesium, sodium	Manganese and zinc	[102,107]
Peppers	Chromium, potassium, zinc, magnesium, sodium	Nickel, Iron, and copper	[102]
Barley	Calcium, copper, zinc		[108]

**Table 8:** Mean concentrations (μg/kg fw) of each element across five vegetables, with values representing means from four retail stores per vegetable. Data adapted from Hadayat et al. [110].

Vegetable	As	Ba	Cd	Cr	Pb
Organic	7.86	410	9.17	44.8	12.1
Conventional	7.29	423	15.3	46.3	17.9

## Data Availability

The original contributions presented in this study are included in the article. Further inquiries can be directed to the corresponding author.
